# Understanding Metal Dynamics Between Cancer Cells and Macrophages: Competition or Synergism?

**DOI:** 10.3389/fonc.2020.00646

**Published:** 2020-04-30

**Authors:** Marina Serra, Amedeo Columbano, Ummi Ammarah, Massimiliano Mazzone, Alessio Menga

**Affiliations:** ^1^Department of Biomedical Sciences, University of Cagliari, Cagliari, Italy; ^2^Laboratory of Tumor Inflammation and Angiogenesis, Center for Cancer Biology (CCB), VIB, Leuven, Belgium; ^3^Department of Molecular Biotechnology and Health Sciences, Molecular Biotechnology Center – MBC, University of Torino, Turin, Italy

**Keywords:** cancer, iron, selenium, copper, zinc, metabolism, crosstalk, TAMs

## Abstract

Metal ions, such as selenium, copper, zinc, and iron are naturally present in the environment (air, drinking water, and food) and are vital for cellular functions at chemical, molecular, and biological levels. These trace elements are involved in various biochemical reactions by acting as cofactors for many enzymes and control important biological processes by binding to the receptors and transcription factors. Moreover, they are essential for the stabilization of the cellular structures and for the maintenance of genome stability. A body of preclinical and clinical evidence indicates that dysregulation of metal homeostasis, both at intracellular and tissue level, contributes to the pathogenesis of many different types of cancer. These trace minerals play a crucial role in preventing or accelerating neoplastic cell transformation and in modulating the inflammatory and pro-tumorigenic response in immune cells, such as macrophages, by controlling a plethora of metabolic reactions. In this context, macrophages and cancer cells interact in different manners and some of these interactions are modulated by availability of metals. The current review discusses the new findings and focuses on the involvement of these micronutrients in metabolic and cellular signaling mechanisms that influence macrophage functions, onset of cancer and its progression. An improved understanding of “metallic” cross-talk between macrophages and cancer cells may pave the way for innovative pharmaceutical or dietary interventions in order to restore the balance of these trace elements and also strengthen the chemotherapeutic treatment.

## Introduction

Tumors occur as a result of the complex interaction between malignant, stromal, immune cells, and vascular system, as these different components communicate with each other via cell–cell contact-dependent mechanisms, soluble messengers and metabolites (1, 2). It is firmly established that the immune system can be reprogrammed by tumor cells to become ineffective, inactivated, or even acquire a tumor promoting phenotype (3). In this special tumor microenvironment the macrophages are particularly abundant and play an important role in tumor development by modulating inflammation, immune suppression, and angiogenesis (2). Many kinds of molecules including growth factors, inflammatory cytokines, chemokines, reactive oxygen, and nitrogen species (ROS and RNS, respectively) from tumor-associated macrophages (TAMs) are involved in the maintenance of a pro-tumorigenic microenvironment and in facilitating metastatic dissemination (3). Recent evidences have highlighted the metabolic signals as important mediators of macrophage function in the crosstalk between cancer and the immune system (4–6). In this metabolic context, cancer patients are characterized by a variety of perturbations in homeostasis of metal ions such as zinc, iron, selenium, and copper both at intratumoral and systemic level (7, 8). A large body of preclinical and clinical studies related to dietary deficiencies, indicates that this metal dysregulation triggers neoplastic transformation of cells and/or reduces anti-tumorigenic functions of immune cells by controlling a plethora of chemical and biological reactions (9). Selenium, copper, zinc, and iron are chemical elements of particular interest given their natural presence in the environment (air, drinking water, and food) and their capacity to stabilize cellular structures, to protect the genome stability, to control metabolic enzymes, receptors, transcription factors at very small concentrations (8, 10). The purpose of this review is to consider the contribution of these trace elements during neoplastic transformation and their involvement in tumor-induced immune evasion (7). Here, we will focus on how metal ions modulate TAMs functions in sustaining immune-suppressive environment that protects tumor cell growth or conversely, how the activity of cancer cells influences TAMs via metallic interplay. New pharmaceutical or dietary intervention strategies with the aim of restoring metal homeostasis, may in the future arise from an improved understanding of “metallic” crosstalk between macrophages and cancer cells.

## Main

### Zinc

Zinc is a vital mineral in many homeostatic mechanisms of the body (11). It activates metabolic enzymes, it is involved in carbonic acid and alcohol formation, it acts as a cofactor for some antioxidant enzymes, such as superoxide dismutase (SOD) and it is essential for the activity of transcription factors and/or proteins regulating gene transcription (9, 10, 12). It is also involved in the signaling pathways of proliferation, differentiation, apoptosis, and cell cycle regulation. Zinc is also crucial for the immune system, since its dyshomeostasis has an effect on proliferation, activation, and apoptosis of immune cells such as monocytes, natural killer-, T-, and B-cells (12, 13). Due to its ubiquitous presence, the immune-modulating properties and the potential ability to alter the function of various important proteins, zinc plays both a direct and an indirect role in the initiation and progression of cancer (14, 15). Moreover, zinc might enhance or decrease the signaling between immune cells and neoplastic cells, by altering membrane structure and receptor expressions (9). The role of zinc homeostasis in regulation of immune system and tumor progression is very complex, depending on its concentration, distribution as well as its temporal pattern (16, 17). Indeed, Zn appears to be protective in some conditions, whereas it is harmful in cases of environmental overexposure (8). Intake of dietary zinc is associated with a reduced risk of gastric, breast, esophageal, prostatic, and colorectal cancer (16), but at plasma concentrations not exceeding 30 μM, in order to avoid immune-suppressive effects (9).

#### Role in Cancer Cells

Many studies support the involvement of two families of metal transporters, namely ZnTs and ZIPs, in different types of cancers (17, 18). The ZnT (SLC30) family reduces cytoplasmic zinc concentrations whereas the ZIP (SLC39) family does the opposite function (19–21). Zn transporters are regulated by the status of zinc itself, hormones, growth factors, as well as cellular redox status (22). Their altered levels of expression or abnormal activities contribute to Zn dyshomeostasis in prostate, pancreatic, breast, and esophageal cancers (16, 17). Ambiguous changes in the expression levels of the zinc efflux transporters (ZnTs) have also been observed during tumorigenesis (21). On one hand, ZnT1 and ZnT2 expression increases in highly aggressive and metastatic basal breast cancer compared to low-invasive luminal, making the cells resistant to Zn toxicity (19, 23, 24). On the other hand, in different cases of more advanced prostatic cancer, ZnT1 and ZnT4 expression (in cytoplasmic vesicles, Golgi apparatus, and plasma membrane) decreases (23–25). Notably, ZnT transporters levels are very low also in pancreatic cancer compared to normal tissues (16, 25), while ZnT7 gene expression is up-regulated in esophageal cancer (17).

Compared to ZnT transporters, many more data are available regarding the association between zinc influx transporters (ZIP) and cancers (11, 19, 24, 25). ZIP1–4 is down-regulated in prostate cancer tissues resulting in low Zn levels in prostate gland (18). Zinc deficiency in prostatic cancer cells is responsible for an increased activity of mitochondrial aconitase and cytochrome c reductase, with consequent high citrate oxidation and respiration, as well as high rate of proliferation and invasiveness (26). In pancreatic cancer tissues all ZIP proteins with the exception of ZIP4 are downregulated, leading to low intracellular Zn concentrations, and increased resistance of the malignant cells to Zn cytotoxic effects (11, 13). In different breast cancer subtypes, zinc distribution, and zinc influx transporter levels show distinct profiles (16, 25). The luminal breast cancer, compared to the basal one, displays the upregulation of several ZIP proteins (ZIP 3, 5, 6, 10, 14) suggesting an increased need of cellular Zn uptake to meet the metabolic demand (25). Intracellular Zn homeostasis is tightly controlled not only by the regulation of the flux across the membranes, but also by buffering of free Zinc by metallothionein and its storage in subcellular organelles, such as vesicles (17, 24). Metallothioneins are small, cysteine-rich, metal-binding proteins which are responsible for maintaining metal homeostasis by acting as metallochaperones, metal donors, and acceptors for enzymes and transcription factors (22). In advanced prostate cancer the expression of metallothioneins, particularly MT1 and MT2, is lower compared to normal tissue and this is associated with increased risk of cancer relapse (21, 24). Conversely, the aggressive basal-like breast cancer exhibits higher levels of metallothioneins than luminal (ER+) and HER2 overexpressing tumors, in order to buffer cytoplasmic Zn and protect the malignant cells from Zn toxicity (21, 25). The behaviors of malignant cancer cells that might appear contradictory in terms of Zn management, within the same type of tumor, must be contextualized to the molecular phenotype of cancer, degree of invasiveness, metastatic potential, and response to therapy. For example, the high invasive basal-like breast cancer tends to throw out and chelate zinc (21, 25), probably with the aim to preserve mitochondrial aconitase and cytochrome c reductase activities and to sustain high oxidative metabolism. Whereas the luminal-like is more inclined to zinc uptake (17), probably in order to avoid an uncontrolled oxidative damage through superoxide dismutase (SOD) activity. The complex interplay between zinc transporters/metallochaperones and zinc signaling is just beginning to be deciphered and requires further investigation. Despite accumulating evidences, whether the accumulation of intracellular zinc pools or the Zn secretion is a “driver” for carcinogenesis is still unclear.

#### Role in Macrophages

The regulation of zinc homeostasis is particularly complex also in immune cells, in particular macrophages. Multiple ZnT/ZIP members are expressed in macrophages, indicating that these transporters have a very important role in physiological conditions (13, 27). Various functions, such as phagocytosis or the secretion of immune-mediating factors can be impaired by deregulation of zinc homeostasis, which ultimately leads to induction or exacerbation of various inflammatory and/or disease processes (22, 28, 29). The relationship between zinc and macrophage functions is very controversial and difficult to figure out. For example, while intracellular zinc levels are induced during early stage of macrophage differentiation whereby they enhance the adhesion process, zinc deficiency inhibits many functions including intracellular killing, cytokine production, and phagocytosis (30, 31). On the one hand, Zn depletion increases monocytes maturation into macrophages (12, 29, 32), on the other hand, it induces apoptosis in macrophages by p53-dependent mechanisms (31, 33). The relationship between zinc and oxidative burst after bacterial infection is also a matter of debate (22, 34). Indeed macrophages utilize two opposite strategies to kill phagocytosed pathogens, (i) by reducing the phagosome zinc content or (ii) by intoxicating them with excess amounts of this metal (12). The relationship between zinc and inflammatory signaling in monocytes/macrophages is still unclear. Chronic zinc deficiency activates the NLRP3 inflammasome and induces the secretion of IL-1β in macrophages, while a short term deficiency inhibits inflammatory activation (29). In addition, LPS treatment of human macrophages in zinc supplemented media increases ZIP8 expression and zinc uptake with consequent C/EBPβ inhibition and the subsequent increase in the pro-inflammatory cytokine response (35).

Zn homeostasis in pro- and anti-inflammatory conditions is also controlled by metallothioneins (MTs). These metal-binding proteins play a fundamental role in macrophage function and in cytokine signaling modulation (12, 22). In response to the pro-inflammatory or M1 cytokines, the macrophages increase Zn uptake by ZIP2 and Zn sequestration by MT1 and MT2, in order to yield a Zn-deficient environment and “steal” this essential element to the pathogen (34, 36). In M2 macrophages polarized with IL-4 or IL-13, MT3 is elevated and suppresses macrophage defenses (22, 36). MT3 renders Zn-pool labile and readily accessible to the pathogen, instead of sequestering it (34, 36). Overall, a lot is yet to be unveiled about the involvement of the metallothionein-Zn axis in immune processes. Indeed, the literature concerning the role of TAMs in maintaining of zinc homeostasis into tumor microenvironment is presently very limited. Ge et al. have highlighted that ZIP8 mediates Zn uptake and that different metallothioneins are induced in TAMs obtained from monocytes treated with melanoma-conditioned medium (30). They concluded that metallothioneins in TAMs sustain high levels of intracellular zinc protecting the cells from stress-induced apoptosis. Overall, the mechanism of how MTs and Zn transporters control TAMs functions in the tumor is limited and further investigation is required.

#### The “Metallic” Cross-Talk Between Macrophages and Cancer Cells

Our understanding of the significance of ZnT, ZIP, and MTs expression within cancer cells and macrophages is still primitive. ZnT, ZIP, and MTs gene expression varies not only in different tumors but also within the tumor. Elevated zinc levels in tumor are characteristic of patients displaying breast, esophageal, lung, and gastrointestinal cancer (16, 17). Zn accumulation in these tumors is in agreement with increased expression of cellular Zn importing proteins compared to normal tissues, suggesting that this mechanism allows them to survive (17). Additionally, liver, kidney, and lung metastasis display higher zinc content in peritumoral tissue than the corresponding normal one or the tumor itself (13).

**Figure 1:**
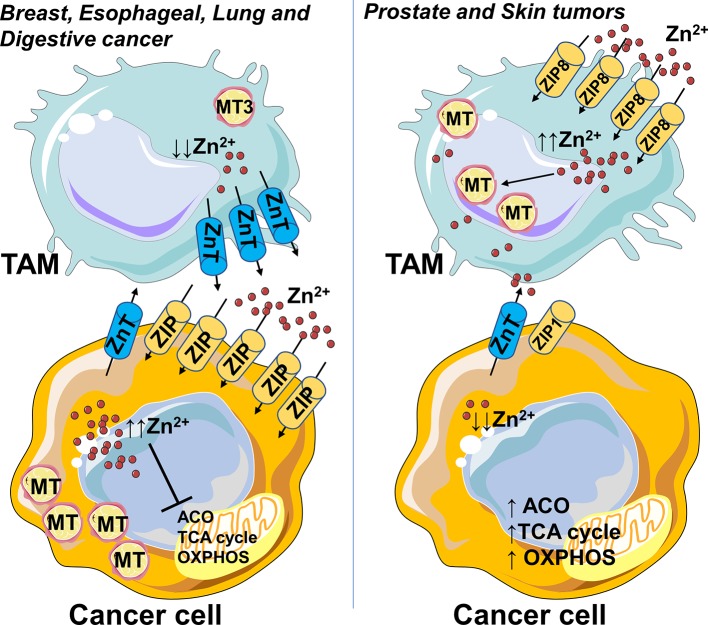
Zinc accumulates in cancer cells or macrophages depending on the type of tumor. Zn accumulation is characteristic of breast, esophageal, lung, and digestive cancer, and correlates with increased expression of cellular Zn importing proteins (ZIP), suggesting that this mechanism allows them to survive. In this context, the M2-like macrophages in the tumor microenvironment could render Zn-pool labile and readily accessible to cancer cells by metallothionein MT3 and ZnT efflux transporters. Unlike other cancer types, prostate and skin tumors have zinc levels lower than normal tissues. Malignant prostate cells lose zinc importer protein ZIP1 and the ability to accumulate zinc, this in turn is associated with a metabolic rewiring, an increased activity of mitochondrial aconitase (ACO) and consequent high citrate oxidation and respiration. In skin tumor, TAMs import zinc and sustain high intracellular levels by upregulation of ZIP8 and metallothioneins, so contributing to protection of cancer cells from Zn toxicity. Higher Zn abduction might be inferred as one of the mechanisms through which TAMs sustain high oxidative metabolism of cancer cells. In parts the figures are based on speculations and have been prepared by assembling in-house built cellular metabolic pathway outlines with a modified and adapted version of BioRender images.

Zinc levels can be directly affected also by the tumor microenvironment. For example, pro-inflammatory mast cells release into cancer microenvironment granules of zinc affecting the cellular response and worsening the prognosis of most cancer patients (13). In this context, one could speculate that M2-like macrophages in the tumor microenvironment render Zn-pool labile and readily accessible to the cancer cells by MT3 and ZnT efflux transporters (Figure 1). Unlike other cancer types, prostate, and skin tumors display lower zinc levels compared to normal tissues (13). Malignant prostate cells are deprived of the ability to accumulate zinc, due to the loss of ZIP1 expression and this is correlated with a metabolic transformation (26). In agreement with Zn “phobic” phenotype of skin tumor, TAMs obtained from monocytes treated with melanoma-conditioned medium, import zinc, and sustain high intracellular levels by upregulating ZIP8 and metallothioneins (30), thus contributing to protection of cancer cells (Figure 1).

Although it is not applicable to any tumor type, it is possible to hypothesize that higher or lower Zn addiction might represent one of the mechanisms by which cancer cells apply a metabolic pressure on the TAMs, leading to immunosuppression, or conversely confer metabolic support to cancer cells.

Evidence of zinc crosstalk between cancer cells and macrophages could unveil a totally new scenario in which novel cellular targets for therapeutic intervention may emerge.

#### Opportunities for Improvement of Cancer Therapy

Several studies suggest the correlation between zinc deficiency and cancer, and some of them support the necessity of zinc supplementation in preventing or treating tumors (9, 37). Yu and co-workers have demonstrated in a murine model of pancreatic cancer that its supplementation via zinc metallochaperones (ZMCs) is able to reactivate quickly and effectively zinc deficient mutants p53 and to recover their wild type transcriptional activities and pro-apoptotic mechanisms (38, 39). These pre-clinical studies might be translated to patients once p53 status of their tumors and zinc-deficient mutations are determined (38). Another way to replenish zinc is by the administration of zinc oxide (ZnO) nanoparticles or sulfate/gluconate formulations. Zn gluconate, used as an adjuvant therapy, has demonstrated its efficacy in stimulating the immune system and in improving the effects of chemotherapy against acute lymphocytic leukemia (Table 1) (44). Zinc sulfate, although at concentrations which exceed those observed in plasma, has revealed cytotoxic effects in colon cancer cells and tumorigenic esophageal epithelial cells (Table 1) (40, 41). Moreover, as well as zinc oxide (ZnO) nanoparticles, zinc sulfate induces a proinflammatory phenotype in a macrophage cell line and in peritoneal macrophages (Table 1) (31, 35), and this may pave the way for innovative TAM-specific agents able to switch the M2-like phenotype toward a tumor-inhibiting M1-like phenotype. On the other hand, excessive zinc supplementation can generate side effects, such as high blood pressure (45). Before starting zinc-based therapy, it would be essential to profile zinc levels in patients and to contextualize them to the molecular phenotype of cancer, histological grading, metastatic potential etc. In luminal-like breast cancer context characterized by zinc requirement, a zinc-based therapy would be counterproductive since it would increase the aggressiveness of the tumor, whereas it would be useful a therapy with strong zinc-chelators. Hashemi and co-workers have demonstrated the cytotoxic power of the cell membrane permeable zinc chelator, N,N,N',N'-tetrakis(2-pyridylmethyl)ethylenediamine (TPEN) and the membrane impermeable zinc chelator, diethylenetriaminepentacetic acid, (DTPA) against breast cancer cells (46).

**Table 1:** Effects of Zinc on cancer cells and macrophages at different concentrations.

**Zn sulfate**	**Effects on cancer cells**	**References**
25 μM	The proliferation curve of cancer cells does not change. No apoptotic neither necrotic effects.	(40)
50 μM	The proliferation curve changes in some cancer cells. Zinc inhibits store-operated calcium entry and intracellular Ca^2+^ level. No apoptotic neither necrotic effects.	(40)
75 μM	Zn significantly affects the growth of cancer cells. No apoptotic neither necrotic effects.	(40)
>75 μM	Zn induces significant growth inhibition ^a^.Many round-shaped and floating dead cells suggest zinc cytotoxicity at these concentrations^b^.	^a^(41)^b^(40)
50 μM	Zn promotes M1 polarization and decreases M2 polarization.	(32)
5, 45, 68, 147 μg/dL	Zinc in co-administration with LPS (100 ng/mL) decreases IL-10 and increases TNFα, IL-8, and IL-6 expression in dose dependent way.	(35)
**ZnO (NPs)**	**Effects on macrophages**	**References**
From 10^−7^ to 10^−3^ μg/mL	NPs induce the polarization of M0 macrophages into M1-like phenotype.	(42)
**Zn gluconate**	**Effects in patients**	**References**
3.18 mg/kg body weight/day	As adjuvant therapy Zn stimulates the immune system and improves the effects of chemotherapy against acute lymphocytic leukemia.	(43)

### Iron

Iron (Fe) is an essential metal for mammalian cells, since Fe-S clusters are the basis of the catalytic activity of many enzymes necessary for heterochromatin stabilization, epigenetic modulations, mitochondrial respiration, TCA cycle etc (47). Iron exhibits a dual effect: on one hand it promotes cell proliferation and growth, on the other hand can induce oxidative damage to DNA, proteins, lipid membranes (i.e., ferroptosis) by production of free oxygen species (ROS) through Fe^2+^-O_2_ reactions and Fenton chemistry (48). Due to iron ability to cause severe DNA strand breaks and modulate epigenome, its dyshomeostasis could be responsible for neoplastic transformation and aggressive tumor cell behavior (48, 49).

#### Role in Cancer Cells

Iron homeostasis and cancer biology are tightly inter-connected, indeed the iron pool is necessary not only for early steps of tumor development, enhanced survival, and proliferation of neoplastic cells, but also for the promotion of metastatic cascade (47, 50). Here, iron is involved in remodeling the extracellular matrix and in the motility of cancer cells (50). Therefore, not surprisingly, elevated levels of Fe have been identified as a risk factor in cancer development and progression (47, 51). In this regard, the role of iron in cancer has been also highlighted by several *in vivo* models (47, 52). In particular, a low-iron diet has been shown to be effective in delaying tumor growth and increasing the survival of mice (53). Malignant tumors display the overexpression of many iron-related genes, and for this reason they compete with liver and spleen for Fe storage, leading to inadequate erythropoiesis and eventually anemia (54). The expression in cancer cells of genes, such as the transferrin receptor (TfR1), ferritin light chain (FTL), and the iron regulatory protein (IRP)-2, is associated with poor prognosis, a higher grade of tumor, and increased resistance to chemotherapy (55, 56). Tumor cells increase iron uptake through the upregulation of divalent metal transporter-1 (DMT1), transferrin/transferrin-receptor (Tf/TfR), and lipocalin-2/lipocalin-2receptor (Lcn-2/Lcn-2R) systems, and its storage by ferritin (FT) heavy chain (FTH) and FTL overexpression (48, 57). The increased iron level in cytosolic compartment supports cellular proliferation and survival functions via cyclinD1/CDK4 overexpression—p21 down regulation and via perturbations in the global histone and DNA methylation (49, 58). At the same time, cancer cells increase mitochondrial uptake of iron via mitoferrin-2 (Mfrn-2) and upregulate frataxin in order to sequester excess iron (that could lead to increased oxidative stress) and deliver it to Fe-S cluster assembly enzyme (ISCU), to allow for Fe-S cluster formation (58–60). To reduce the risk of iron overloading-dependent lipid peroxidation (that leads to non-apoptotic form of cell death known as ferroptosis) cancer cells rely on the selenoprotein glutathione dependent peroxidase 4 (GPX4) activity, which decreases intracellular radicals and protects mitochondrial metabolism from ROS-induced membrane damage (61, 62). Iron accumulation in cancer cells is also exacerbated by deregulation of iron exporter ferroportin1 (FPN1). In invasive tumor areas, FPN expression is lower compared to normal tissue and inversely correlated with patient survival and disease outcome (48, 63, 64). The expression of FPN is regulated by hepcidin, a protein linked to cancer driven inflammation which induces internalization and degradation of FPN upon its binding (48, 65). In cancer patients, elevated levels of hepcidin allow to control local tumor iron efflux by an autocrine/paracrine regulatory mechanism (48, 66). Given the complex network of iron regulatory genes in cancer cells a better understanding of their regulation and interplay is necessary.

#### Role in Macrophages

Immune cells such as macrophages and T cells require iron to shape their phenotype and determine their responses (67, 68). Macrophages have a very important role to play in iron recycling from the RBCs. In spleen and liver, macrophages swallow up senescent RBCs and heme oxygenases (HO-1 and HO-2) catabolize the heme. The iron resulting from this process is then stored either in ferritin (FT) or exported via ferroportin (FPN) (69, 70). The FT^high^ and FPN1^low^ pro-inflammatory macrophages display an iron sequestering phenotype characterized by iron withdrawal, restriction and storage (71, 72). Furthermore, these kind of macrophages enhance the uptake of iron-containing heme clusters and the expression of heme oxygenase 1 (HO-1) in order to recycle heme-iron and increase labile iron pool (LIP) (71, 72). It is worthy of interest that excess amounts of heme or iron in hemorrhagic tumor areas, caused by hemolytic red blood cells (RBCs), shift the pro-tumoral macrophage phenotype toward a pro-inflammatory and anti-tumoral one, which in turn exacerbates tissue damage (67). On the other hand, the FT^low^ FPN1^high^ anti-inflammatory macrophages are predisposed to iron export and redistribution to the extracellular space, supporting the demand of surrounding cells (47, 71, 72). It has been widely demonstrated, both *in vitro* and *in vivo*, that anti-inflammatory macrophages TAMs adopt a strong iron-release phenotype that contributes to tumor cell proliferation and growth (57). In some cases, the inability of their FPN to export iron, due to the high levels of local hepcidin, is bypassed thanks to the increased expression of high-affinity iron-binding protein lipocalin-2 (Lcn-2) (47, 57). Since tumors demand an excess of iron, further investigations on TAM heterogeneity and iron plasticity are urgently needed.

#### The “Metallic” Cross-Talk Between Macrophages and Cancer Cells

**Figure 2:**
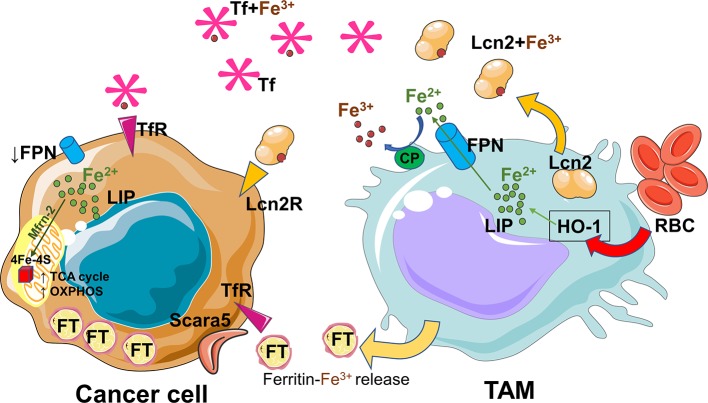
Iron recycled and released by TAMs sustains the cancer progression. The pro-tumoral/anti-inflammatory macrophages adopt an iron-export phenotype. The released iron is absorbed by cancer cells, driven to mitochondria via mitoferrin-2 (Mfrn-2) and delivered to Fe-S clusters proteins essential for mitochondrial respiration and the activities of TCA cycle enzymes. TAMs rely on uptake of senescent red blood cells (RBC) and on the activity of heme oxygenase 1 (HO-1) in order to recycle heme-iron and increase labile iron pool (LIP). Iron can be released via ferroportin (FPN) and after ceruloplasmin (CP) activity loaded onto circulating transferrin (Tf) for its uptake by cancer cells via the transferrin receptor (TfR). Alternatively, TAMs can use lipocalin-2 system or release ferritin to transfer iron. During cancer progression, tumor cells increase iron storage by ferritin (FT) overexpression, and iron uptake by upregulation of the ferritin light chain binding protein Scara5, transferrin-receptor (TfR) and lipocalin-2receptor (Lcn-2R). At the same time cancer cells decrease iron export by deregulation of ferroportin1 (FPN1). The figures have been prepared by assembling in-house built cellular metabolic pathway outlines with a modified and adapted version of BioRender images.

In the tumor microenvironment both tumor and immune cells compete for nutrients and metal elements such as iron (47, 48, 73). As mentioned before, iron plays also an important role in cancer development (48). Several evidences firmly established the concept of iron crosstalk between cancer cells and macrophages (47, 74). During early stages of carcinogenesis, pro-inflammatory cytokines and the exposure to hemolytic red blood cells (RBCs) shift the macrophages toward an iron loaded phenotype (67). Consequently, it is not surprising that cancer and macrophage cells compete for iron uptake in the tumor microenvironment. Later, the pro-tumoral/anti-inflammatory macrophages adopt an iron-release phenotype and donate iron to the tumor microenvironment to support cancer progression (75) (Figure 2). Iron can be released via FPN and loaded onto circulating Tf for its uptake by cancer cells via the TfR. Alternatively, TAMs can rely on lipocalin-2 or ferritin release to transfer iron (47, 74, 76). To date, it is not known if iron removal from tumor microenvironment by iron-demanding cancer cells could be responsible for a shift toward a pro-tumoral and anti-inflammatory M2-like phenotype, as it happens in a renal inflammatory context (77). A better understanding of how iron controls crosstalk between macrophages and cancer cells requires further investigation.

#### Opportunities for Improvement of Cancer Therapy

Considering the role of iron in regulating immune and cancer cells functions, therapies targeting iron metabolism are urgently needed. Cancer cells are iron influx dependent, and in line with this concept the application of iron chelators, dietetic iron depletion, and interference with the hepcidin pathway represents a first intervention strategy *in vivo* and *in vitro* (47, 78, 79). Various iron chelators able to inhibit cancer cell growth and modulate global histone and DNA methylation have been employed for iron overload disorders (49, 51). But to date, none has obtained approval for the cancer treatment, due to unfavorable pharmacokinetics and lack of selectivity (48). At the same time, several drugs and antibodies that interfere with hepcidin expression or activation have been developed with promising effects, but unfortunately, the lack of long-term follow-up studies in patients does not allow to predict their efficacy and safety (80–82). Moreover, some FPN stabilizers are being developed, in order to reactivate iron efflux from tumor cells (48, 81). However, since the pathways that regulates the hepcidin-FPN axis are complex, further studies are needed. Another emerging possibility is to target excess iron in tumor cells through induction of ferroptosis (48, 83, 84). In this regard, GSH depletion by erastin and inactivation of GPX4 activity by FDA approved alkylating antineoplastic compound altretamine (hexamethylmelamine) have shown their efficacy as ferroptosis-inducer (61, 62, 85, 86). It is worthy of interest that ferroptotic secretome released from dying cancer cells is able to promote the recruitment of immune cells and support an M1-type immune microenvironment (87). To date, there is an increasing reliance on the use of micro/nanoparticles in cancer therapy. The treatment of tumor-bearing mice with iron microparticles has resulted in M1-like iron-loaded macrophages and net tumor suppression (67, 88). Another type of iron nanoparticle, the FDA approved ferumoxytol, has been shown to reduce the tumor growth and polarizing the macrophages toward M1 like phenotype in mammary, liver, and lung cancers (89). Additional *in vivo* studies and clinical trials are required for many of these compounds to elucidate their specific anticancer properties and their efficacy. Moreover, it would be useful to correlate iron levels in serum and tumors with the molecular phenotype of cancer, in order to choose the best therapy.

### Copper

Copper is an essential transition metal required for fundamental metabolic processes, but it can be toxic if present in excess (90, 91). As catalytic cofactor of many enzymes, it is involved in the mitochondrial electron transport chain (cytochrome c oxidase), in the detoxification of reactive oxygen species (superoxide dismutase 1 and 3), in the conversion of hydroperoxides into hydroxides (glutathione peroxidase), in melanin formation (tyrosinase), and in “ferroxidation” (ceruloplasmin) (91). Copper ions are also fundamental for proteins involved in cell signaling pathways, cell differentiation and death, and for enzymes involved in nervous system physiology. This metal ion plays a crucial role in the development and maintenance of immune function (29, 92). Indeed copper-deficient patients display decreased numbers of myeloid precursors in the bone marrow and susceptibility to infections (29, 93).

The recommended daily intake of copper in healthy adults is 0.9 mg/day (94).

A reduced intake of copper causes neutropenia, anemia, hypotonia, deterioration of the nervous system, neurodegenerative disorders, and severe intellectual disabilities. Whereas the overload of copper, mainly in the liver, brain, and kidney, results in redox copper toxicity (e.g., liver cirrhosis) (91, 95). Various studies suggest a strong involvement of altered copper and cupro-proteins levels in cancer (96, 97). Copper has the ability to catalyze redox reactions and during its dysregulation reactive oxygen species are generated so excessively that act as precursors for neoplastic transformation and metastasis formation (91, 98). Many types of cancer (brain, multiple myeloma, acute lymphoblastic leukemia, lung, reticulum cell sarcoma, cervical, breast, and stomach cancer) show increased intratumoral levels and/or altered overall distribution of copper (97).

#### Role in Cancer Cells

An analysis of the human copper proteome in 18 different tumor types has revealed several copper genes like CTR1, ATOX1, ATP7B, COX17 to be up-regulated (91). The reduced copper (including the dietary pool) is transported inside the cells via CTR1, a high affinity membrane copper transporter. The increased copper flow via CTR1 is followed by loading onto copper chaperone ATOX1, which acts as a copper-dependent transcription factor promoting the transcription of cyclin D1 and prompting cell replication (91, 99). Furthermore, copper binds to copper chaperones like COX17 and SCO2, which deliver it to mitochondria and to target proteins involved in trans Golgi network, including ATP7A, and ATP7B (100). Since copper is essential for the activity of cytochrome c oxidase (Cox), mitochondria rely on the phosphate carrier SLC25A3 for its uptake (101), and on labile copper pool in endoplasmic reticulum as additional source (91, 102). The mitochondrial phosphate carrier SLC25A3 has been associated with chronic myeloid leukemia progression and might play a role in copper imbalance (103). MEK1 being a copper-binding protein has led to the hypothesis that this metal ion is involved in the RAS-RAF-MEK-ERK pathway, required for cell proliferation, and tumorigenesis (104). Copper not only binds to proteins directly involved in cancer progression, but also indirectly modulates their expression or activation. Copper inhibits prolyl hydroxylase thus stabilizing HIF-1α and increasing the transcription of various angiogenic genes (e.g., ceruloplasmin and VEGF) (105) and genes involved in the epithelial to mesenchymal transition (e.g., LOX) (91, 106). The copper-dependent enzyme LOX catalyzes the cross-linking of collagen and elastin in the extracellular matrix (ECM) and interacts with MEMO1 (Mediator of cell Motility 1), another copper-dependent redox enzyme (107). MEMO1 is involved in cell migration through modulation of the cytoskeleton and formation of adhesion sites. Furthermore, copper ions activate the endothelial Nitric Oxide Synthetase (eNOS), thus increasing the production of the vasodilator nitric oxide (NO) (108). Other studies are required to unveil the mechanisms by which these proteins within the cell are loaded with copper. The dysregulation of these protein functions could be the priming for processes such as, creation of pre-metastatic niches, escape of immune defense, and angiogenesis. Understanding the mechanism of these genes and protein may open up exciting avenues for developing them as potential cancer therapeutic targets.

#### Role in Macrophages

Copper is an essential element for immunomodulatory functions (29). As a component of the SOD enzyme, which catalyzes the production of H_2_O_2_ from superoxide, it sustains the activity of neutrophils and monocytes, and regulates macrophage antimicrobial functions by contributing to ROS-dependent killing capacity (29, 109). Indeed its deficiency leads to a defective respiratory burst, impaired phagocytosis, and killing ability, with consequent susceptibility to recurrent pulmonary and urinary infections as well as septicaemia (29, 110, 111). Macrophages activated with proinflammatory cytokines (IFNγ and TNFα) and LPS show increased copper uptake via CTR1, increased copper accumulation within the phagosomes due to bactericidal Fenton reactions, and finally increased ceruloplasmin activity (112). The copper-containing ferroxidase ceruloplasmin promotes iron export via FPN, thus starving intracellular bacteria of this essential element (29, 113). Furthermore, M1-like macrophages display also an increased copper transport to the mitochondria via COX17 for energy production, to SOD1 for antioxidant defense or to Atp7a for protein synthesis (29, 112). The literature on the role of copper in modulating M2-like macrophages and/or in sustaining TAMs function into tumor microenvironment is absent.

#### The “Metallic” Cross-Talk Between Macrophages and Cancer Cells

**Figure 3:**
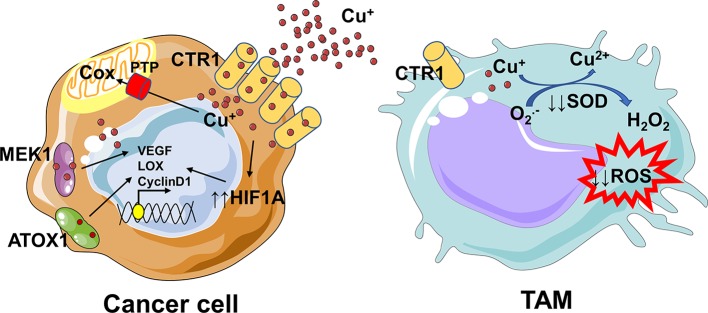
Copper addiction of cancer cells could prevent the pro-inflammatory phenotype of macrophages. The increased copper flow via CTR1 is followed by loading onto the copper chaperone ATOX1, which acts as a copper-dependent transcription factor promoting cyclin D1 expression and cell replication. Since copper is essential for the activity of proteins, like cytochrome c oxidase (Cox), involved in the mitochondrial electron transport chain, mitochondria rely on the phosphate carrier SLC25A3 (PTP) for its uptake. Copper not only binds to proteins directly involved in cancer progression, such as MEK1, but also indirectly modulates their expression or activation. Copper inhibits prolyl hydroxylase thus stabilizing HIF-1α and increasing the transcription of several angiogenic genes (e.g., ceruloplasmin and VEGF) and genes involved in the epithelial to mesenchymal transition (e.g., LOX). Copper is essential for sustaining the pro-inflammatory phenotype of macrophages; indeed, as a component of the SOD enzyme which catalyzes the production of H_2_O_2_ from superoxide, it contributes to the ROS-dependent killing capacity of macrophages. The removal of copper from microenvironment by cancer cells might drive the polarization of TAMs toward a pro-tumoral M2-like phenotype. In parts the figures are based on speculations and have been prepared by assembling in-house built cellular metabolic pathway outlines with a modified and adapted version of BioRender images.

There are not evidences on the copper crosstalk between cancer cells and macrophages, thus in this context we can only speculate. Several studies suggest a strong copper addiction of cancer cells (114, 115), that probably deprives TAMs of this essential element. Since copper is essential for sustaining the pro-inflammatory phenotype of macrophages (29, 113), its removal from tumor microenvironment could be responsible for a shift toward a pro-tumoral M2-like phenotype and for an immunosuppressive environment (Figure 3). Overall, our understanding of how copper controls TAMs-cancer cells interplay requires further investigation, with the aim to plan in the future a better dietary intervention or to find novel targets and innovative therapeutic agents.

#### Opportunities to Improve Cancer Therapy

The strong connection between copper and tumor development, as well as metastization has encouraged scientists to design and synthesize new copper-complexing agents to be used in chemotherapy with lower side effects (79, 91). The copper-binding compounds used as anticancer agents are divided in two groups: copper chelators, which sequester copper ions from cells, and copper ionophores, which vehicle copper inside cells increasing its intracellular levels and priming cytotoxic effects through multiple pathways (116, 117). The copper complexing species tetrathiomolybdate (TTM), disulfiram, and clioquinol have been employed in clinical trials, but only TTM has given the most promising results (117). In the latest years, the fact that copper is a limiting factor for multiple phases of tumor progression, has led the scientists to the identification of plant based natural molecules with chelating properties, able to exert antitumoral effects or improve the efficacy of already known drugs, with low side effects (91, 97). These compounds in the presence of copper act as pro-oxidants and produce reactive oxygen species so excessively to induce DNA degradation (91, 118). The effects of copper, copper oxide nanoparticles, and copper chelate have been evaluated not only on cancer cells but also on macrophages (88, 119). Chatterjee et al., discovered a novel copper chelate, copper N-(2-hydroxy acetophenone) glycinate (CuNG), able to reprogram TAMs in a proinflammatory type which in turn converts Treg and Th2 cells in anti-tumorigenic Th1 cells (120–122). This compound triggers in TAMs ROS-mediated activation of MAPKs and ERK1/2 pathways which lead to upregulation of IL-12 and simultaneous downregulation of TGF-β and IL-10 production (121). We may speculate on a bivalent role of these redox-active compounds like CuNG in a clinical approach. The sustained generation of ROS on the one hand would induce apoptosis of cancer cells, on the other hand would trigger proimmunogenic macrophages.

### Selenium

The metal ion selenium (Se) plays important role in different biological processes which are mediated by almost 25 selenoproteins (123). As a cofactor for antioxidant enzymes, it exhibits anti-inflammatory properties and inhibits oxidative damage as well as DNA alterations (9). Moreover, selenium homeostasis supports the innate and adaptive immune functions; indeed its deficiency is associated with T cells and NK cells dysfunction and with a reduced number of lymphocytes in both thymus and bursa (9). Selenium is generally transported by selenoprotein P (SEPP1) and its mutations and/or haplo insufficiency increases genomic instability and risk of cancer (124, 125). Indeed, populations with low Se intake are exposed to higher risk of cancer development and its supplementation in suboptimal doses enhances immune responses to prevent cancer growth, reduce relapse, and cancer-specific mortality (9, 14). However, supra-nutritional doses do not confer protection against cancer and are associated with toxicity (123).

#### Role in Cancer Cells

A recent study on SELENOP (SEPP1) has led to the identification of several single nucleotide polymorphisms (SNPs) which decrease the expression or function of this metal in various tumor types, including hepatocellular carcinomas, gastric adenocarcinomas, colorectal cancer, and prostate cancer (126–128). SEPP1 is one of the few selenoproteins (SePs) able to incorporate selenium, be secreted into the plasma, be absorbed by the other tissues, and degraded to free selenium for synthesis of other SePs (129). SEPP1 loss induces an oxidative stress which, on one hand, can increase DNA damage and favor tumor initiation, on the other, can promote cancer cell cytotoxicity (127, 128, 130). However, SEPP1 expression is not universally down regulated in all tumor types. Indeed, SEPP1 upregulation has been observed in metastatic melanoma and poorly differentiated prostate cancer (128, 131). In cancer cells having high basal levels of oxidative stress, increased expression of SEPP1 can protect from cytotoxic effects and also lead to increased tumor development, proliferation, and resistance to chemotherapy (128). Selenium by lowering ROS production/accumulation not only prevents DNA oxidation but also activates mechanisms that stimulate mitochondrial biogenesis, preserve mitochondrial membrane potential, and sustain metabolic performance (132).

The glutathione peroxidases GPxs (1,2,3,4, and 6) constitute some of the most thoroughly studied SePs, because of their role in oxidative stress and their contribution to tumorigenesis (133). These proteins have antioxidant properties and catalyze hydroperoxide reduction by using glutathione (GSH) as a reductant. GPx1 expression is decreased in many tumor types and its overexpression, both *in vitro* and *in vivo*, has been found to reduce the growth of cancer cells and carry out a protective role (128, 134, 135). On the other hand, GPx1 expression has been linked to higher tumor number and growth rate, as well as to chemo/radio resistance (136). Like GPx1, GPx2 appears to have a pro-tumorigenic role in esophagus and liver, whereas it exhibits an anti-inflammatory role in colon context. Indeed, its deficiency has been linked to colitis-associated tumorigenesis (137, 138). Among the glutathione peroxidases, GPx3 is the only one clearly acting as a tumor suppressor. In tumor cells, GPx3 is often a target of hypermethylation and its downregulation is associated with bad prognosis and chemoresistance in several types of tumor (128, 139). Other selenoproteins with a critical role in maintaining redox balance and in controlling the multiple stages of tumor progression are the thioredoxin reductases (TrxRs). They are selenium responsive elements able to trigger antioxidant defense mechanisms in response to selenium supplementation (128, 140). Several *in vitro* and *in vivo* studies agree that TrxRs can inhibit tumor growth by extinguishing oxidative damage and DNA alterations, especially in the context of inflammatory-driven cancers. However, in tumor cells with higher basal levels of oxidative stress, these TrxRs can increase the resistance to apoptosis and even to chemotherapy (128, 141). Much work still needs to be done to characterize SePs in tumorigenesis context and to identify and understand the mechanisms by which they influence neoplastic transformation. The contradictory behavior of malignant cancer cells in terms of selenium management, needs to be deepened and contextualized to type of tissue, molecular phenotype, and degree of invasiveness, in order to determine the benefits or not of selenium supplementation. Selenium as regulator of cell redox balance can have different effects, depending on whether or not the tumor is inflammatory-driven.

#### Role in Macrophages

A great body of evidence has extensively highlighted the role of selenium in the modulation of immune processes, particularly in macrophages (124). Studies on macrophage-specific knockout of selenocysteine (Sec) tRNA gene (Trsp), have demonstrated that selenoproteins drive their polarization from a pro-inflammatory toward an anti-inflammatory phenotype, which aids in the resolution of inflammation and wound healing (124, 142, 143). In particular, loss of Trsp leads to a decrease in M2 macrophage markers, a corresponding increase in M1 macrophage markers, an altered regulation in extracellular matrix-related gene expression and a diminished migration of macrophages in a protein gel matrix (124, 144, 145).

This phenotypic switch is combined with changes in cellular metabolism, particularly of arachidonic acid (146). Selenium in macrophages, by differential regulation of expression of mPGES1, TXAS, and H-PGDS, plays an important role in bioactive oxylipids synthesis, such as cyclopentenone prostaglandins (CyPGs) (145, 146). In presence of selenium, the arachidonic acid is metabolized to 15d-PGJ_2_, which negatively affects pro-inflammatory signal transduction pathways (146). Vunta et al., demonstrated that selenium deficiency in mice exacerbates the LPS-mediated infiltration of macrophages into the lungs and also that selenium reintegration in macrophages leads to a significant decrease in LPS-induced expression of cyclooxygenase-2 (COX-2) and tumor necrosis factor-a (TNF-a) (146). Furthermore, other studies have associated the ability of selenoproteins to downregulate the expression of pro-inflammatory genes and polarize the macrophages toward an M2 phenotype with the inhibition of histone and non-histone acetylation, the activation of PPARγ and the degradation of pro-inflammatory PGE2 (145, 147). Experiments of gene expression have revealed that SELENOP (SEPP1) is one of the most upregulated genes in the M2 macrophage phenotype (128, 148). Moreover, Solinas et al., have found SELENOP (SEPP1) upregulated 95-fold at the transcript level in macrophages polarized by cancer cells conditioned media (149). Despite the lack of experimental evidence, it is possible to hypothesize that the increased SELENOP in M2 macrophages may offset the loss of SELENOP in cancer cells and support metastasis by supplying it in a paracrine manner (150). On the other hand, Barrett et al., have highlighted a shift toward M2 phenotype stimulated by IFN-γ and LPS (M1) or IL-13 (M2) in bone marrow derived macrophages isolated from Sepp1^+/−^ mice (124). In agreement with these results, other studies have associated the selenium deficiency to the loss of GPxs and phagocytic activities of macrophages (M1 feature) toward transformed cells (133, 145, 150).

Development of mouse models lacking selenoproteins in macrophages has paved the way for understanding immune modulatory properties of these proteins (143, 144). However, the role of individual selenoproteins in this process is yet to be investigated properly. Based on the *in vivo* studies, selenium supplementation is essential to effectively resolve inflammation in most instances (145). Thus, it remains to understand if also selenocompounds may play a protective role.

#### The “Metallic” Cross-Talk Between Macrophages and Cancer Cells

**Figure 4:**
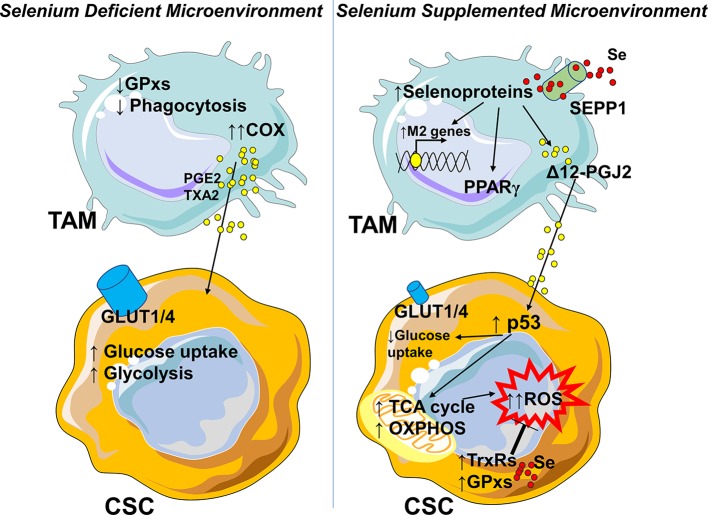
Selenium availability in the tumor microenvironment influences the phenotype of macrophages. In a Se-deficient microenvironment TAMs lose glutathione dependent peroxidases (GPxs) and phagocytic activities (M1-like feature) toward transformed cells and produce PGE2 and TXA2 from arachidonic acid by cyclooxygenase (COX) supporting the highly glycolytic cancer stem cells (CSC). In the presence of selenium, TAMs rely on selenium importer SEPP1, and increase the selenoproteins which in turn polarize the macrophages toward an M2-like phenotype, with activation of PPARγ, degradation of pro-inflammatory PGE2, and production of Δ12-PGJ2. Δ12-PGJ2 activates the tumor suppressor protein p53, which in turn upregulates the TCA cycle and oxidative phosphorylation and lowers glucose uptake by the cells. As a compensatory mechanism the antioxidant machinery (selenium dependent thioredoxin reductases TrxRs and glutathione peroxidases GPxs) is enhanced but the increase is not sufficient to control ROS production. In a Se-rich microenvironment, its lower uptake might be assumed as one of the mechanisms through which neoplastic cells modulate M2-like macrophages and/or sustain TAMs function. In parts the figures are based on speculations and have been prepared by assembling in-house built cellular metabolic pathway outlines with a modified and adapted version of BioRender images.

The role of selenium in the cross-talk between macrophages and cancer cells has been demonstrated only in leukemia disease. In a Se-deficient microenvironment TAMs produce mostly PGE2 and TXA2 from arachidonic acid *via* the COX pathway, supporting the highly glycolytic cancer stem cells (CSC) (145) (Figure 4). Following Se-supplementation, selenoproteins affect the production of Δ12-PGJ2 in the M2 macrophage. Δ12-PGJ2 released by macrophages activates in cancer stem cells the tumor suppressor protein p53, which in turn upregulates the TCA cycle, oxidative phosphorylation, and lowers glucose uptake (145, 151, 152). As a compensatory mechanism, the antioxidant machinery is increased, although it is not sufficient to control ROS production and to avoid apoptosis (145) (Figure 4). Also in this case, a better understanding of how selenium controls TAMs-cancer cells interplay will require further investigation. To date, without adequate experimental evidences, we may only speculate that the absence of selenium transporter SEPP1 in tumors and its increased expression in M2-like macrophages, tip selenium balance toward an immunosuppressive and pro-tumorigenic microenvironment. One may suggest that lower Se uptake by cancer cells is one of the mechanisms by which they drive the macrophage shift toward a proangiogenic, immunosuppressive, and pro-tumoral function.

#### Opportunities for Improvement of Cancer Therapy

Selenium supplementation is an attractive and achievable way to decrease cancer incidence, since selenium compounds are generally cheap and, at appropriate doses, safe (153, 154). Various studies have identified many classes of natural as well as synthetic organoselenium compounds which act as cytotoxic agents, and the research is ongoing for identifying more such compounds (154–157). Keeping in mind the immunomodulatory function of selenium, selenium nanoparticles (SeNPs) have been synthesized (158, 159). SeNPs have potential to decrease tumor cell proliferation, drive the anti-tumor function of TAMs, and in virtue of their properties, be used for imaging diagnosis and cancer therapy with low costs and negligible side effects (154, 158, 159). An impressive number of *in vitro* and *in vivo* studies clearly confirms the scarce toxicity of selenium compounds as monotherapy and in combination with classical chemotherapy (154). Furthermore, they also seem to increase the therapeutic potential of other drugs and reduce their side effects. However, to date the antiproliferative and proapoptotic properties of selenite, selenium amino acids, and other selenium compounds have not been confirmed by clinical trials (155, 156). Since supra-nutritional doses do not confer protection against cancer, on the contrary are associated with toxicity, before choosing a selenium-based therapy, it would be essential to profile serum and tumoral levels of metal ion, and to contextualize them to type of tissue, molecular phenotype, histological grading, metastatic potential, and chemosensitivity. In tumors characterized by high basal levels of oxidative stress, resistance to ROS-, and chemotherapy-mediated apoptosis, a selenium-based therapy would be counterproductive since it would increase tumor development and proliferation. More focused *in vivo* studies and additional clinical trials are necessary.

## Concluding Remarks

The effects of zinc, iron, selenium, and copper on cancer cells and TAMs (in supplementation or deficiency context) vary with concentration and tumor type. To sum up: heme iron intake and high serum levels of iron are associated with increased risk of breast and liver cancer (160); copper overload causes liver, lung, urinary, stomach, and cervical cancer; zinc poisoning or deficiency are associated with breast, lung, gastric, colon, and prostatic cancer; lower selenium intake increases liver, gastric, colon, and prostatic cancer incidence. In most cases these ions have been studied individually and their combined contribution to cancer progression has been totally overlooked or not well understood. In cancer growth and immune escape context, it is very important to consider also the relationship and balance between these metal ions inside the tumoral tissue. For example, a lower Zn/Fe ratio in the malignant prostatic tissue is correlated with poor prognosis and increased resistance to chemotherapy (161). In this case, zinc deficiency and iron overload combine their metabolic effects to increase citrate oxidation and mitochondrial activity in cancer cells and support their energy status. Also the Se/Zn balance plays an important role in onset of cancer. When the selenium is in excess compared to zinc, the metallothionein system is dysregulated, thereby producing p53 loss of function and DNA integrity reduction (162). Moreover, the results of some studies suggest that there is a close relationship between Cu and Fe in macrophages. Indeed highly toxic ferrous iron, as result of decreased ceruloplasmin expression/activity and copper deficiency, accumulates in macrophages leading to severe dysfunction (163). How the different ions contribute collectively to all steps of carcinogenesis and immune suppression remains to be described. The few observations made in co-culture systems and small animal models need to be amplified, extended to ion-ion interactions and carefully translated to the human setting. Wisely designed clinical trials are necessary to establish how the neoplastic cells influence TAMs functions or conversely, by controlling metal ions flux. A better understanding of the metal dynamics by which cancer remodels its microenvironment, may aid the discovery of innovative therapies able to more effectively kill tumor cells, or at least limit tumor progression and metastatic dissemination.

## Author Contributions

This manuscript was conceived jointly by all authors. MS wrote the first draft of the manuscript. AM designed the paper and figures. MS, AC, UA, MM, and AM have revised and approved the final manuscript.

## Conflict of Interest

The authors declare that the research was conducted in the absence of any commercial or financial relationships that could be construed as a potential conflict of interest. The handling Editor declared a past co-authorship with one of the authors MM.
